# European Option Based on Least-Squares Method under Non-Extensive Statistical Mechanics

**DOI:** 10.3390/e21100933

**Published:** 2019-09-25

**Authors:** Limin Liu, Yingying Cui

**Affiliations:** College of Mathematics and Information Science, Henan Normal University, Xinxiang 453007, China; cuiyingying0430@163.com

**Keywords:** European call option, non-extensive statistical mechanics, least-square method, error analysis

## Abstract

This paper is devoted to the study of the pricing of European options under a non-Gaussian model. This model follows a non-extensive statistical mechanics which can better describe the fractal characteristics of price movement in the financial market. Moreover, we present a simple but precise least-square method for approximation and obtain a closed-form solution of the price of European options. The advantages of this technique are illustrated by numerical simulation, which shows that the least-squares method is better compared with Borland’s two methods in 2002 and 2004.

## 1. Introduction

Since the famous Black–Scholes [1] model was put forward in 1973, many scholars have begun to deal with financial investment problems in terms of the Black–Scholes model. Wang [2] studied the Black–Scholes option pricing model based on the dynamic investment strategy. Glazyrina [3] showed another derivation of how the normal approximation of the binomial distribution leads to the Black–Scholes formula from the binomial option pricing model. Ulyah et al., [4] proposed a new pricing model for short-term foreign stock options by Black–Scholes theory.

It is well-known that the Black–Scholes model assumed that stock prices follow a logarithmic normal distribution and have independent increments. However, Lo [5] and Lux [6] have confirmed that the returns of financial assets have some fractal properties, such as the sharp peak, fat tail, and long-term memory. Therefore, the hypothesis in Black–Scholes model is inconsistent with the empirical results. In addition, the underestimation of the options based on Black–Scholes model results in the volatility smile curve. Hence, many scholars have begun to correct the Black–Scholes model. Merton [7] proposed a jump model to describe the intermittent fluctuation of price. Hull [8] introduced the stochastic volatility model. Necula [9] established a new price model by using fractional Brownian motion. Since these models can well depict the characteristics of long memory or sharp peak and fat tail of the return distribution, Hubalek et al., [10], Xiao [11] and Gu [12] popularized the application of these models in the financial field.

In 1988, Tsallis [13] put forward the theory of non-extensive Tsallis entropy. Then Borland [14] studied the fractal characteristics of Tsallis distribution from the perspective of microdynamics. Liu [15] systematically discussed the self-similarity, non-Markovian and long dependence of this distribution. Since the non-extensive Tsallis has the fractal characteristics, it can well model the distributions of asset returns of many financial assets. In 2002, Borland [16] began to apply Tsallis theory to the pricing problem and achieved a quiet better result. Later, some scholars used this theory to asset portfolio, option pricing and other aspects and achieved better results [17,18,19,20,21].

In the process of option pricing, the key issue is to solve the integral $\int_{0}^{T}f(\Omega (t),t)dt$. To address this problem, Borland transformed this integral into a function in terms of Ω(T) and *T* by substituting Ω(t) with $\sqrt{\frac{\beta (T)}{\beta (t)}}\Omega \left(T\right)$ for the reason that $\sqrt{\frac{\beta (T)}{\beta (t)}}\Omega \left(T\right)$ and Ω(t) have identical distribution. Zhao [22] extended this method to the average geometric Asian option pricing problem. In 2004, Borland [23] used the Feynman–Kac equation and $Pad\overset{´}{e}$ ansatz operator to reconsider the integral approximation and got the price formula of European call option. Then Wang [24] extended this method to the average geometric Asian option pricing problem. However, the above scholars did not discuss the effectiveness of the two approximations of Borland. In this paper, we used a new least-square method to reconsider the approximation problem and discussed the effectiveness of our method, by which to promote the wide application of Tsallis theory in the financial field.

The rest of the paper is organized as follows. In Section 2, we establish the asset price model by non-extensive statistics theory. In Section 3, we use the least-square method to solve the integral approximation problem. In Section 4, we deduce the closed solution of European option which is similar to the Black–Scholes formula. In Section 5, the effectiveness of our method is discussed. In Section 6, we summarize our paper.

## 2. Price Model Based on the Non-Extensive Statistics

This section considers a price model which is same as Borland’s model in [16,23]. The characteristic of this model is that the noise of stock price fluctuation is a non-Gaussian process.

Suppose that there are two kinds of assets in the market. One is the risk-free bond with a price satisfying the equation below:

(1) $$\left\{\begin{array}{c} dB(t)=rB(t)dt,\\ B(0)=1, \end{array}\right.$$

where *r* is risk-free rate, B(t) is the bond price. The other asset in the market is the stock, its price S(t) follows

(2) dS(t)=μS(t)dt+σS(t)dΩ(t),

with

(3) $$d\Omega \left(t\right)=P{\left(\Omega \left(t\right),t\right)}^{\frac{1-q}{2}}dW\left(t\right),$$

where Ω(t) is a non-Gaussian noise source, W(t) is a standard Brownian motion defined on the probability space $\left(\Omega ,{\left\{F_{t}\right\}}_{t\geq 0},P\right)$. P(Ω(t),t) comes from the non-extensive entropy theory in statistical physics, which refers to the probability distribution when Tsallis entropy is maximized under the constraints of regularization and normalization.

P(Ω(t),t) satisfy the following nonlinear Fokker-Planck equation:

(4) $$\frac{\partial P(\Omega \left(t\right),t|\Omega \left(t^{\prime }\right),t^{\prime })}{\partial t}=\frac{1}{2}\frac{\partial^{2}}{\partial \Omega {\left(t\right)}^{2}}P^{2-q}\left(\Omega \left(t\right),t|\Omega \left(t^{\prime }\right),t^{\prime }\right),$$

where *q* is the non-extensive parameter. Plastino and Plastino [25] have proved that the conditional probability in Equation (4) has a form as

(5) $$P\left(\Omega \left(t\right),t|\Omega \left(t^{\prime }\right),t^{\prime }\right)=\frac{1}{Z(t)}{\left(1-\beta \left(t\right)\left(1-q\right){\left(\Omega \left(t\right)-\Omega \left(t^{\prime }\right)\right)}^{2}\right)}^{\frac{1}{1-q}},$$

with

$$\beta \left(t\right)=c^{\frac{1-q}{3-q}}{\left[\left(2-q\right)\left(3-q\right)\left(t-t^{\prime }\right)\right]}^{-\frac{2}{3-q}},$$

$$Z\left(t\right)={\left[\left(2-q\right)\left(3-q\right)c\left(t-t^{\prime }\right)\right]}^{\frac{1}{3-q}},$$

$$c=\frac{\pi }{q-1}\frac{\Gamma^{2}\left(\frac{1}{q-1}-\frac{1}{2}\right)}{\Gamma^{2}\left(\frac{1}{q-1}\right)},$$

where Γ(·) is the Gamma function. In addition, as q→1, Ω(t) becomes a Gaussian process. Let $t^{\prime }=0$, Ω(0)=0, we can get the following Tsallis distribution [26]:

(6) $$P\left(\Omega \left(t\right),t\right)=\frac{1}{Z(t)}{\left[1-\beta \left(t\right)\left(1-q\right)\Omega {\left(t\right)}^{2}\right]}^{\frac{1}{1-q}}.$$

From Equation (6), it is easily to verify that the mean function of Ω(t) is zero and the variance function is

(7) $$Var\left[\Omega \left(t\right)\right]=\frac{1}{\left(5-3q)\beta (t\right)}.$$

Since we are only interested in the process with finite variance, it is reasonable to assume $1\leq q<\frac{5}{3}$.

When there is no arbitrage opportunity in the market, for a risk-neutral investor, the expected return of the asset in the future must be the risk-free rate. In other words, the fair market value should discount accordingly in the risk-neutral framework at the risk-free rate. The following Theorem deduces the formula of stock price based on Tsallis distribution in the fair market, which is the reorganization and restatement of Borland [16].

**Theorem** **1.** *In the risk-neutral market, the stock price S(t) satisfying the stochastic differential Equation (2) is*

(8) $$\begin{array}{c} S\left(t\right)=S\left(0\right)\exp\{\sigma \Omega \left(t\right)+rt-\frac{\sigma^{2}}{2}\delta \left(\Omega \left(t\right),t\right)\}, \end{array}$$

*where S(0) is the price at time 0, and*

(9) $$\begin{array}{c} \delta \left(\Omega \left(t\right),t\right)=\int_{0}^{t}{\left(\frac{1}{Z(s)}\right)}^{1-q}\left[1-\beta \left(s\right)\left(1-q\right)\Omega {\left(s\right)}^{2}\right]ds. \end{array}$$

**Proof.**  Define the discounted stock price J(t) is

(10) $$J\left(t\right)=e^{-rt}S\left(t\right).$$

Since there is generally no arbitrage opportunity in a risk-neutral financial market, Equation (10) is required to be a martingale process. According to Girasanov’s theorem, we can find an equivalent measure Q corresponding to another noise term $\tilde{W}\left(t\right)$ that transforms Equation (10) into a martingale. Let

(11) $$d\tilde{W}\left(t\right)=\kappa dt+dW\left(t\right),$$

and

$$\kappa =\frac{\mu -r}{\sigma P^{\frac{1-q}{2}}},$$

where

$$P^{\frac{1-q}{2}}=P{\left(\Omega \left(t\right),t\right)}^{\frac{1-q}{2}}.$$

Then the new measure Q is related to original P by the following Radon–Nikodym derivative

$$\frac{dQ}{dP}{|}_{F_{t}}=\exp\left(-\int_{0}^{t}\kappa dW\left(t\right)-\frac{1}{2}\int_{0}^{t}\kappa^{2}ds\right).$$

Using $It\hat{o}$ formula on Equation (10), we obtain

(12) $$\begin{array}{c} dJ\left(t\right)=\left(\mu -r\right)J\left(t\right)dt+\sigma P^{\frac{1-q}{2}}J\left(t\right)dW\left(t\right). \end{array}$$

Inserting (11) into (12) yields

(13) $$\begin{array}{cc} dJ(t) & =\left(\mu -r\right)J\left(t\right)dt+\sigma P^{\frac{1-q}{2}}J\left(t\right)dW\left(t\right)\\ \; & =\sigma J\left(t\right)P^{\frac{1-q}{2}}\left(\frac{\mu -r}{\sigma P\frac{1-q}{2}}dt+dW\left(t\right)\right)\\ \; & =\sigma J\left(t\right)P^{\frac{1-q}{2}}d\tilde{W}\left(t\right). \end{array}$$

Thus, J(t) becomes a martingale process.

Using $It\hat{o}$ formula we get

(14) $$d\ln S\left(t\right)=rdt-\frac{1}{2}\sigma^{2}P^{1-q}dt+\sigma P^{\frac{1-q}{2}}d\tilde{W}\left(t\right),$$

which implies that

$$\begin{array}{c} S\left(t\right)=S\left(0\right)\exp\{\sigma \Omega \left(t\right)+rt-\frac{\sigma^{2}}{2}\delta \left(\Omega \left(t\right),t\right)\}, \end{array}$$

where δ(Ω(t),t) is defined by (9).  ☐

## 3. Approximate Solution Based on Least-Square Method

Since integral δ(Ω(t),t) has the form of $\int_{0}^{t}f(\Omega (s),s)ds$, it cannot be solved directly. In this section, we use the least-square method (LSM) to find a simple regression equation to approximate this integral.

As mentioned in Equation (7), the stochastic process Ω(t) has the mean of 0 and the variance of $\frac{1}{\left(5-3q)\beta (t\right)}$. Suppose the standardized variable of Ω(t) is $\Omega^{*}$ expressed as

$$\begin{array}{c} \Omega^{*}=\frac{\Omega (t)}{\sqrt{\frac{1}{\left(5-3q)\beta (t\right)}}}, \end{array}$$

then $\Omega^{*}$ follows a standardized Tsallis distribution with the mean of 0 and the variance of 1. Similarly, for any time *s*, it is easily to obtain

$$\begin{array}{c} {\tilde{\Omega }}^{*}=\frac{\Omega (s)}{\sqrt{\frac{1}{\left(5-3q)\beta (s\right)}}}. \end{array}$$

This means that $\Omega^{*}$ and ${\tilde{\Omega }}^{*}$ are identically distributed, i.e.

(15) $$\Omega \left(s\right)\overset{d}{=}\sqrt{\frac{\beta (t)}{\beta (s)}}\Omega \left(t\right).$$

It can be clearly seen that if we use the Ω(t) to substitute the Ω(s) in Equation (9), then the form of δ(Ω(t),t) can be transformed more simple so that we can solve. Following this intuition, inserting Equation (15) into Equation (9) yields

(16) $$\delta \left(\Omega \left(t\right),t\right)=\frac{1}{2}\left(3-q\right){\left(\left(2-q\right)\left(3-q\right)\varepsilon \right)}^{\frac{q-1}{3-q}}t^{\frac{2}{3-q}}\left(1-\left(1-q\right)\beta \left(t\right)\Omega {\left(t\right)}^{2}\right),$$

By observing Equation (16), we find δ is a function about $t^{\frac{2}{3-q}}$ and $\Omega {\left(t\right)}^{2}$. Hence, the basis functions are $t^{\frac{2}{3-q}}$, $\Omega {\left(t\right)}^{2}$ and $t^{\frac{2}{3-q}}\cdot \Omega {\left(t\right)}^{2}$. Suppose the approximate expression of δ(Ω(t),t) is

(17) $$\hat{\delta }\left(\Omega \left(t\right),t\right)=d_{1}\left(q\right)\cdot t^{\frac{2}{3-q}}+d_{2}\left(q\right)\cdot \Omega {\left(t\right)}^{2}+d_{3}\left(q\right)\cdot t^{\frac{2}{3-q}}\Omega {\left(t\right)}^{2}+d_{4}\left(q\right).$$

**Definition** **1.** *The estimated error E between $\hat{\delta }$ and δ is*

(18) $$E=E_{P}\left[{\left(\delta \left(\Omega \left(t\right),t\right)-\hat{\delta }\left(\Omega \left(t\right),t\right)\right)}^{2}\right],$$

*where $E_{P}\left[\cdot \right]$ represents the mean under measure P.*

Borland [16] in 2002 mapped Ω(s) onto the Ω(t) by Equation (15) and derived an approximate expression for δ(Ω(t),t).

**Remark** **1.** *The approximation of δ(Ω(t),t) derived by Borland in 2002 is*

(19) $$\begin{array}{c} \hat{\delta_{1}}\left(\Omega \left(t\right),t\right)=\frac{1}{2}\left(3-q\right){\left(\left(2-q\right)\left(3-q\right)c\right)}^{\frac{q-1}{3-q}}t^{\frac{2}{3-q}}\\ -\frac{\left(3-q)(1-q\right)}{2}\beta \left(t\right){\left(\left(2-q\right)\left(3-q\right)c\right)}^{\frac{q-1}{3-q}}t^{\frac{2}{3-q}}\Omega {\left(t\right)}^{2}. \end{array}$$

By comparing with Equation (17), it can be seen that $d_{j}$ in Equation (17) take the following form respectively,

$$\begin{array}{cc} d_{1} & =\frac{1}{2}\left(3-q\right){\left(\left(2-q\right)\left(3-q\right)c\right)}^{\frac{q-1}{3-q}},\\ d_{2} & =0,\\ d_{3} & =-\frac{\left(3-q)(1-q\right)}{2}\beta \left(t\right){\left(\left(2-q\right)\left(3-q\right)c\right)}^{\frac{q-1}{3-q}},\\ d_{4} & =0. \end{array}$$

However, the disadvantage of the method (15) is that Ω(t) is a random process not a simple distribution, the substitution of (15) will cause a large deviation of the approximation of δ(Ω(t),t). Then Borland proposed a new method in 2004 taken in [23], in which he used the Feynman–Kac equation and $Pad\overset{´}{e}$ ansatz to reconsider the approximate of δ(Ω(t),t) and got another approximation result.

**Remark** **2.** *The approximation of δ(Ω(t),t) derived by Borland in 2004 is*

(20) $$\hat{\delta_{2}}\left(\Omega \left(t\right),t\right)={\left(\left(2-q\right)\left(3-q\right)c\right)}^{\frac{q-1}{3-q}}\left(\frac{3-q}{2}-\frac{\left(1-q)(3-q\right)}{2(9-5q)}\right)t^{\frac{2}{3-q}}-\frac{1-q}{9-5q}\Omega {\left(t\right)}^{2}$$

Similar comparison with Equation (17), it can be seen that the $d_{j}$ in (17) are

$$\begin{array}{cc} d_{1} & ={\left(\left(2-q\right)\left(3-q\right)c\right)}^{\frac{q-1}{3-q}}\left(\frac{3-q}{2}-\frac{\left(1-q)(3-q\right)}{2(9-5q)}\right),\\ d_{2} & =\frac{1-q}{9-5q},\\ d_{3} & =0,\\ d_{4} & =0. \end{array}$$

## 4. European Option Price

In this section, the approximate function $\hat{\delta }\left(\Omega \left(t\right),t\right)$ is used to solve the pricing problem of European options.

We first consider the pricing of European call options. A European call option has a payoff of

(21) $$C_{T}=\max\left[S\left(T\right)-K,0\right],$$

where S(T) represents the price of a risky asset at maturity *T* and *K* is the strike price. Under the measure Q, the price of this option is

(22) $$\begin{array}{cc} C_{0} & =E_{Q}\left[e^{-rT}C\right]\\ \; & =E_{Q}\left[e^{-rT}S\left(T\right)I_{\left\{S(T)>K\right\}}\right]-E_{Q}\left[e^{-rT}KI_{\left\{S(T)>K\right\}}\right]\\ \; & =A_{1}-A_{2}, \end{array}$$

with

$$\begin{array}{cc} A_{1} & =E_{Q}\left[e^{-rT}S\left(T\right)I_{\left\{S(T)>K\right\}}\right],\\ A_{2} & =E_{Q}\left[e^{-rT}KI_{\left\{S(T)>K\right\}}\right], \end{array}$$

where $E_{Q}\left[\cdot \right]$ represents the mean value under measure Q and $I_{\left\{S(T)>K\right\}}$ is an indicator function.

**Theorem** **2.** *The price of the European call option is given by*

(23) $$\begin{array}{c} C_{0}=S\left(0\right)M_{q}\left(\gamma_{1},\gamma_{2}\right)-Ke^{-rT}N_{q}\left(\gamma_{1},\gamma_{2}\right), \end{array}$$

*where*

$$\begin{array}{cc} \; & M_{q}\left(\gamma_{1},\gamma_{2}\right)=e^{-rT}\left(\int_{\gamma_{1}}^{\gamma_{2}}\exp\left\{\sigma \Omega \left(T\right)-\frac{\sigma^{2}}{2}\hat{\delta }\left(\Omega \left(T\right),T\right)\right\}P\left(\Omega \left(T\right),T\right)d\Omega \left(T\right)\right),\\ \; & N_{q}\left(\gamma_{1},\gamma_{2}\right)=\int_{\gamma_{1}}^{\gamma_{2}}P\left(\Omega \left(T\right),T\right)d\Omega \left(T\right),\\ \; & P\left(\Omega \left(T\right),T\right)=\frac{1}{Z(T)}{\left[1-\beta \left(T\right)\left(1-q\right)\Omega {\left(T\right)}^{2}\right]}^{\frac{1}{1-q}},\\ \; & \hat{\delta }\left(\Omega \left(T\right),T\right)=d_{1}\left(q\right)\cdot T^{\frac{2}{3-q}}+d_{2}\left(q\right)\cdot \Omega {\left(T\right)}^{2}+d_{3}\left(q\right)\cdot T^{\frac{2}{3-q}}\Omega \left(T\right)+d_{4}\left(q\right),\\ \; & \gamma_{1,2}=\frac{-a_{2}\pm \sqrt{a_{2}^{2}-4a_{1}\cdot a_{3}}}{2a_{1}a_{2}},\\ \; & a_{1}=-\frac{\sigma^{2}}{2}\left(d_{2}\left(q\right)+d_{3}\left(q\right)T^{\frac{2}{3-q}}\right),\\ \; & a_{2}=\sigma ,\\ \; & a_{3}=rT-\frac{\sigma^{2}}{2}d_{1}\left(q\right)T^{\frac{2}{3-q}}-\frac{\sigma^{2}}{2}d_{4}\left(q\right)-\ln\frac{K}{S(0)}. \end{array}$$

**Proof.**  By Theorem 1, the approximate expression of the stock price is

(24) $$\begin{array}{cc} S(T) & =S\left(0\right)\exp\{\sigma \Omega \left(T\right)+rT-\frac{\sigma^{2}}{2}\hat{\delta }\left(\Omega \left(T\right),T\right)\}\\ \; & =S\left(0\right)\exp\{\sigma \Omega \left(T\right)+rT-\frac{\sigma^{2}}{2}(d_{1}\left(q\right)\cdot T^{\frac{2}{3-q}}\\ \; & +d_{2}\left(q\right)\cdot \Omega {\left(T\right)}^{2}+d_{3}\left(q\right)\cdot T^{\frac{2}{3-q}}\Omega \left(T\right)+d_{4}\left(q\right))\}. \end{array}$$

To calculate $A_{1}$ and $A_{2}$ in Equation (22), we should first solve the inequality {S(T)>K}. Using Equation (24) yields

(25) $$\begin{array}{cc} \; & S\left(0\right)\exp\{\sigma \Omega \left(T\right)+rT-\frac{\sigma^{2}}{2}(d_{1}\left(q\right)\cdot T^{\frac{2}{3-q}}\\ \; & +d_{2}\left(q\right)\cdot \Omega {\left(T\right)}^{2}+d_{3}\left(q\right)\cdot T^{\frac{2}{3-q}}\Omega \left(T\right)+d_{4}\left(q\right))\}>K. \end{array}$$

Taking logarithms of both sides of Equation (25) obtain

(26) $$\begin{array}{c} a_{1}\Omega {\left(T\right)}^{2}+a_{2}\Omega \left(T\right)+a_{3}>0, \end{array}$$

where

$$\begin{array}{cc} a_{1} & =-\frac{\sigma^{2}}{2}\left(d_{2}\left(q\right)+d_{3}\left(q\right)T^{\frac{2}{3-q}}\right),\\ a_{2} & =\sigma ,\\ a_{3} & =rT-\frac{\sigma^{2}}{2}d_{1}\left(q\right)T^{\frac{2}{3-q}}-\frac{\sigma^{2}}{2}d_{4}\left(q\right)-\ln\frac{K}{S(0)}. \end{array}$$

The quadratic equation

(27) $$\begin{array}{c} a_{1}\Omega {\left(T\right)}^{2}+a_{2}\Omega \left(T\right)+a_{3}=0 \end{array}$$

has the discriminant as

$$\begin{array}{cc} △ & =a_{2}^{2}-4a_{1}\cdot a_{3}\\ \; & =\sigma^{2}+2\times \left(\sigma^{2}\left(d_{2}\left(q\right)+d_{3}\left(q\right)T^{\frac{2}{3-q}}\right)\right)\\ \; & \times (rT-\frac{\sigma^{2}}{2}d_{1}\left(q\right)T^{\frac{2}{3-q}}-\frac{\sigma^{2}}{2}d_{4}\left(q\right)-\ln\frac{K}{S(0)}). \end{array}$$

Assuming that △>0, there are two roots of quadratic Equation (27)

$$\begin{array}{c} \gamma_{1,2}=\frac{-a_{2}\pm \sqrt{a_{2}^{2}-4a_{1}\cdot a_{3}}}{2a_{1}a_{2}}. \end{array}$$

Therefore, we get the solution set of {S(T)>K} as $\Omega \left(T\right)\in \left(\gamma_{1},\gamma_{2}\right)$. Substituting the roots into $A_{1}$ and $A_{2}$, we finally get

$$\begin{array}{cc} A_{1} & =E_{Q}\left[e^{-rT}S\left(T\right)\cdot I_{S(T)>K}\right]=\int_{\gamma_{1}}^{\gamma_{2}}e^{-rT}\cdot S\left(T\right)\cdot P\left(\Omega \left(T\right),T\right)d\Omega \left(T\right)\\ \; & =e^{-rT}S\left(0\right)(\int_{\gamma_{1}}^{\gamma_{2}}\exp\left\{\sigma \Omega \left(T\right)+rT-\frac{\sigma^{2}}{2}\hat{\delta }\left(\Omega \left(T\right),T\right)\right\}P\left(\Omega \left(T\right),T\right)d\Omega \left(T\right))\\ \; & =S\left(0\right)M_{q}\left(\gamma_{1},\gamma_{2}\right).\\ A_{2} & =E_{Q}\left[e^{-rT}K\cdot I_{S(T)>K}\right]=\int_{\gamma_{1}}^{\gamma_{2}}e^{-rT}\cdot K\cdot P\left(\Omega \left(T\right),T\right)d\Omega \left(T\right)\\ \; & =Ke^{-rT}N_{q}\left(\gamma_{1},\gamma_{2}\right). \end{array}$$

where

$$\begin{array}{cc} \; & M_{q}\left(\gamma_{1},\gamma_{2}\right)=e^{-rT}\left(\int_{\gamma_{1}}^{\gamma_{2}}\exp\left\{\sigma \Omega \left(T\right)-\frac{\sigma^{2}}{2}\hat{\delta }\left(\Omega \left(T\right),T\right)\right\}P\left(\Omega \left(T\right),T\right)d\Omega \left(T\right)\right),\\ \; & N_{q}\left(\gamma_{1},\gamma_{2}\right)=\int_{\gamma_{1}}^{\gamma_{2}}P\left(\Omega \left(T\right),T\right)d\Omega \left(T\right). \end{array}$$

Hence the value for a European call option is

$$\begin{array}{cc} C_{0} & =A_{1}-A_{2}\\ \; & =S\left(0\right)M_{q}\left(\gamma_{1},\gamma_{2}\right)-Ke^{-rT}N_{q}\left(\gamma_{1},\gamma_{2}\right). \end{array}$$

 ☐

**Corollary** **1.** *According to the parity formula of European call-put option, the price of European put option is*

$$\begin{array}{c} P_{0}=S\left(0\right)\left(M_{q}\left(\gamma_{1},\gamma_{2}\right)-1\right)-Ke^{-rT}\left(N_{q}\left(\gamma_{1},\gamma_{2}\right)-1\right). \end{array}$$

## 5. Numerical Results

In this section, we use numerical simulation to compare LSM method and Borland’s methods from two aspects, one is the values of integral approximation, the other is the option prices.

To compare the three methods, we first generate the paths of this process by using the following Euler’s iterative formula:

(28) $$\Omega \left(t_{i}\right)-\Omega \left(t_{i-1}\right)={\left(\frac{1}{Z(t_{i-1})}\right)}^{\frac{1-q}{2}}{\left(1-\beta \left(t_{i-1}\right)\left(1-q\right)\Omega {\left(t_{i-1}\right)}^{2}\right)}^{\frac{1}{2}}\left(W\left(t_{i}\right)-W\left(t_{i-1}\right)\right).$$

Moreover, the values of δ(Ω(t),t) is calculate by rectangle integral principle that are expressed as

(29) $$\begin{array}{cc} \delta (\Omega (t),t) & =\sum_{i=0}^{n-1}\{\frac{1}{2}(Z{\left(t_{i}\right)}^{\left(q-1\right)}\left[1-\beta \left(t_{i}\right)\left(1-q\right)\Omega {\left(t_{i}\right)}^{2}\right]\\ \; & +Z{\left(t_{i+1}\right)}^{\left(q-1\right)}\left[1-\beta \left(t_{i+1}\right)\left(1-q\right)\Omega {\left(t_{i+1}\right)}^{2}\right])\times \left(t_{i+1}-t_{i}\right)\}, \end{array}$$

where *n* in Equations (28) and (29) represents the number of nodes in the path. $t_{i}$ refers to the time point corresponding to each node.

The steps of the numerical simulation mainly include three aspects represented as follows.

Step 1. Use the Euler’s iterative formula (28) to generate the path of Ω(t).

Step 2. Use the rectangle integral principle (29) to generate δ(Ω(t),t).

Step 3. Perform the multiple nonlinear regression based on LSM via (17) to get the regression parameters.

Use 1000 paths, and each path has 100 nodes. The following Table 1 reports the corresponding simulation results of regression parameter values. As can be seen from Table 1, the value of each parameter $d_{j}$ varies with the difference of *q*.

**Figure 1:**
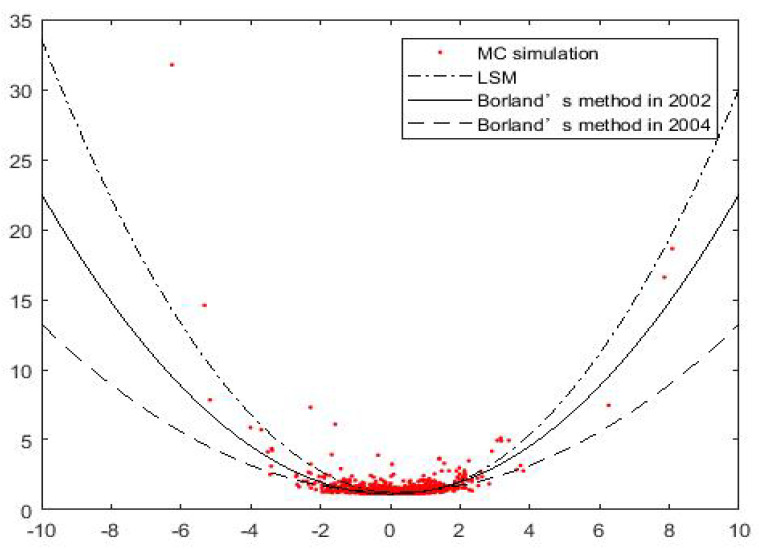
The horizontal axis is Ω(T), and the vertical axis is the corresponding values of δ. Scatter points are obtained by Monte Carlo simulation.

Assuming q=1.3, T=1, we get the following Figure 1 which depicts the quadratic relationship between $\hat{\delta }$ and Ω(T). Scatter points represent Monte Carlo simulation results. It can be seen that the approximate functions obtained by the three methods are close to each other in the place with dense scatter points, but differ greatly in the place with few scatter points. The approximation based on LSM method can better fit the sparse points distributed at both ends implying that our method is much closer to the results of Monte Carlo.

The following Table 2 is the results of errors of δ calculated by LSM and the other two methods, respectively. From Table 2, we can see that the values of error vary with the values of *q*. It is obviously to find that the $\hat{\delta }$ calculated with the LSM has the smallest errors in the three methods.

Before examining the numerical simulation of the call option price by three method, we first give the price formula of European call option deduced by Borland in 2002 and 2004, respectively.

**Remark** **3.** *The price of European call option based on Borland’s method in 2002 is*

(30) $$\begin{array}{cc} C_{0} & =S\left(0\right)\int_{\xi_{1}}^{\xi_{2}}\exp\left\{\sigma \Omega \left(T\right)-\frac{\sigma^{2}}{2}\hat{\delta_{1}}\left(\Omega \left(T\right),T\right)P\left(\Omega \left(T\right),T\right)d\Omega \left(T\right)\right\}\\ \; & -Ke^{-rT}\int_{\xi_{1}}^{\xi_{2}}P\left(\Omega \left(T\right),T\right)d\Omega \left(T\right), \end{array}$$

*where $\hat{\delta_{1}}$ is same as Equation (19) and $\xi_{1,2}=\frac{s_{1,2}}{\sigma \sqrt{\frac{1}{\left(5-3q)\beta (T\right)}}}$, $s_{1,2}$ take the form as*

(31) $$\begin{array}{cc} s_{1,2} & =\frac{-1}{\rho (T)(1-q)\sigma \beta (T)}\\ \; & \pm \left[\frac{1}{\rho \left(T\right)T^{\frac{2}{3-q}}{\left(1-q\right)}^{2}\sigma^{2}\beta {\left(T\right)}^{2}}-\frac{2}{\left(1-q\right)\rho \left(T\right)\sigma^{2}\beta \left(T\right)}\right]\\ \; & \times (rT+\ln\frac{S(0)}{K}-\frac{\sigma^{2}}{2}\rho \left(T\right)), \end{array}$$

*with*

$$\begin{array}{c} \rho \left(t\right)=\frac{1}{2}\left(3-q\right){\left(\left(2-q\right)\left(3-q\right)\varepsilon \right)}^{\frac{q-1}{3-q}}\cdot t^{\frac{2}{3-q}}. \end{array}$$

**Remark** **4.** *The price based on Borland’s method in 2004 is*

(32) $$\begin{array}{cc} C_{0} & =S\left(0\right)\int_{\pi_{1}}^{\infty }\exp\{\sigma \Omega \left(T\right)-\frac{\sigma^{2}}{3-q}(1-\left(q-1\right)(b_{1}\left(T\right)\\ \; & +b_{2}\left(T\Omega {\left(T\right)}^{2}\right)))P\left(\Omega \left(T\right),T\right)d\Omega \left(T\right)\}\\ \; & -Ke^{-rT}\int_{\pi_{1}}^{\infty }P\left(\Omega \left(T\right),T\right)d\Omega \left(T\right), \end{array}$$

*where $\pi_{1}=\frac{\sigma T}{2}$ and $b_{1}$, $b_{2}$ are defined by*

$$b_{1}\left(t\right)={\left(\left(2-q\right)\left(3-q\right)\varepsilon \right)}^{\frac{q-1}{3-q}}\cdot \frac{3-q}{2(9-5q)}\cdot t^{\frac{2}{3-q}},$$

*and*

$$b_{2}\left(t\right)=\frac{1}{9-5q}.$$

Letting S(0)=50, r=0.04, σ=0.2, q=1.3 and T=0.8, we use the price Formulas (23), (30) and (32) to calculate the option prices and absolute errors based on three method respectively. The absolute errors represent the absolute values of the difference of prices between each method and the Monte Carlo simulation. In Table 3, as can be seen, the price based on LSM is closest to the result of Monte Carlo simulation. Therefore, the obtained option price based on LSM is better than the two methods of Borland’s.

**Figure 2:**
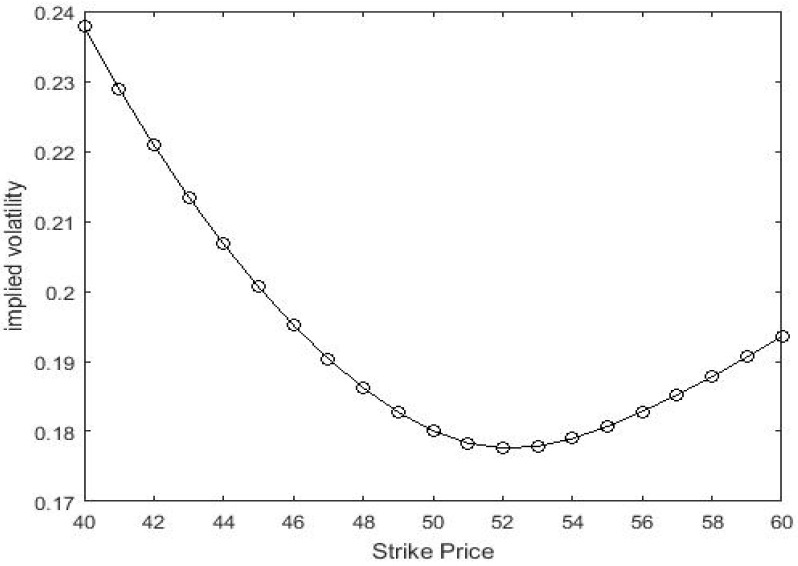
The horizontal axis is *K*, and the vertical axis is the corresponding values of implied volatilities.

Figure 2 below is an implied volatility curve based on LSM model, which is plotted as a function of strike price *K*. We substitute the prices calculated by Equation (23) (q=1.3, σ=0.2, S(0)=50, T = 0.8 and r=0.04) into Blake-Scholes model to back out the implicit volatility. Obviously, these implied fluctuations form a smiling shape, very similar to the shape implied by real market data. Moreover, the downward sloping smile curve reproduces well-known systematic features of the volatility smile that appears when using the standard Black–Scholes to price real options.

## 6. Conclusions

This paper mainly solves the pricing problem of European options with a non-Gaussian model. Considering the characteristics of abnormal diffusion of financial asset prices, we apply the non-extended Tsallis entropy theory with this characteristic to the price movement model of assets. Since the integral $\int_{0}^{t}f(\Omega (s),s)ds$ in the price formula cannot be calculated, we use LSM to evaluate. Finally, the numerical simulation results show that the least-square method is better than that of Borland’s in 2002 and 2004. 

## Figures and Tables

**Table 1:** Parameter values at different values of *q*.

qValue	$d_{1}\left(q\right)$	$d_{2}\left(q\right)$	$d_{3}\left(q\right)$	$d_{4}\left(q\right)$
q=1.1	1.0723	0.0250	0.0067	0.0015
q=1.2	1.5556	0.0614	0.0234	0.0059
q=1.3	1.2401	0.1049	0.0904	0.0234
q=1.4	1.1963	0.0576	0.4463	0.1468
q=1.5	0.4591	−0.2623	1.5190	1.0991

**Table 2:** Error analysis of the δ for three methods.

qValue	Borland(2002)	Borland(2004)	LSM
q=1.1	0.0274	0.0156	0.0154
q=1.2	0.2058	0.1514	0.1433
q=1.3	1.4325	1.4236	1.2196
q=1.4	25.1968	29.8205	20.6674
q=1.5	2179.1	2334.4	1316.6

**Table 3:** Error of option price for three methods.

Strike	Monte	Pricesof	Pricesof	Pricesof	Errorsof	Errorsof	Errorsof
Price	Carlo	Borland(2002)	Borland(2004)	LSM	Borland(2002)	Borland(2004)	LSM
45	7.4546	7.4900	7.4916	7.4522	0.0354	0.0371	0.0023
46	6.6749	6.7078	6.7108	6.6722	0.0329	0.0359	0.0027
47	5.9325	5.9628	5.9677	5.9298	0.0304	0.0352	0.0026
48	5.2331	5.2606	5.2677	5.2307	0.0275	0.0345	0.0025
49	4.5820	4.6061	4.6157	4.5796	0.0242	0.0337	0.0024
50	3.9860	4.0066	4.0191	3.9840	0.0206	0.0331	0.0020
51	3.4506	3.4673	3.4829	3.4490	0.0167	0.0323	0.0017
52	2.9799	2.9926	3.0113	2.9786	0.0127	0.0314	0.0013
53	2.5729	2.5819	2.6036	2.5721	0.0090	0.0308	0.0008
54	2.2250	2.2302	2.2548	2.2244	0.0052	0.0298	0.0006
55	1.9286	1.9306	1.9576	1.9282	0.0020	0.0290	0.0004

## References

[1] Black F., Scholes M. (1973). The pricing of options and corporate liabilities. J. Polit. Econ..

[2] Wang X., Wang L. (2007). Study on Black–Scholes stock option pricing model based on dynamic investment strategy. Int. J. Innov. Comput. Inf. Control..

[3] Glazyrina A., Melnikov A. (2016). Bernstein’s inequalities and their extensions for getting the Black–Scholes option pricing formula. Stat. Probab. Lett..

[4] Ulyah S.M., Lin X.C., Miao D.W. (2017). Pricing short-dated foreign equity options with a bivariate jump-diffusion model with correlated fat-tailed jumps. Financ. Res. Lett..

[5] Lo A. (1991). Long term memory in stock market prices. Econometrica.

[6] Lux T. (1996). The stable Paretian hypothesis and the frequency of large returns: An examination of major German stocks. Appl. Financ. Econ..

[7] Merton R.C. (1976). Option pricing when underlying returns are discontinuous. J. Financ. Econ..

[8] Hull J.C. (2006). Options, Futures, and Other Derivatives.

[9] Necula C. (2002). Option Pricing in a Fractional Brownian Motion Environment. https://papers.ssrn.com/sol3/papers.cfm?abstract_id=1286833.

[10] Hubalek F., Keller-Ressel M., Sgarra C. (2017). Geometric asian option pricing in general affine stochastic volatility models with jumps. Quant. Financ..

[11] Xiao W., Zhang W., Zhang X., Wang Y.L. (2010). Pricing currency options in a fractional Brownian motion with jumps. Econ. Model..

[12] Gu H., Liang J., Zhang Y. (2012). Time-changed geometric fractional Brownian motion and option pricing with transaction costs. Phys. A Stat. Mech. Appl..

[13] Tsallis C. (1988). Possible Generalization of Boltzmann-Gibbs statistics. J. Stat. Phys..

[14] Borland L. (1998). Microscopic dynamics of the nonlinear fokker-planck equation: A phenomenological model. Phys. Rev. E.

[15] Liu L., Cui Y., Xu J., Li C., Gao Q. (2019). The Non-Markovian Property of q-Gaussian Process.

[16] Borland L. (2002). A theory of non-Gaussian option pricing. Quant. Financ..

[17] Ferrari D., Paterlini S. (2010). Efficient and Robust Estimation for Financial Returns: An Approach Based on Q-Entropy. https://papers.ssrn.com/sol3/papers.cfm?abstract_id=1906819.

[18] Li S., He J., Song K. (2016). Network entropies of the chinese financial market. Entropy.

[19] Wang Y., Li D., Wei J. (2017). Pricing of Power European Options Based on Tsallis Entropy and O-U Process under Stochastic Interest Rate. J. Zhengzhou Univ..

[20] Devi S. (2017). Financial market dynamics: Superdiffusive or not?. J. Stat. Mech. Theory Exp..

[21] Liu L., Zhang L., Fan S. (2018). Optimal investment problem under non-extensive statistical mechanics. Comput. Math. Appl..

[22] Zhao P., Zhou B., Wang J. (2018). Non-Gaussian closed form solutions for geometric average Asian options in the framework of non-extensive statistical mechanics. Entropy.

[23] Borland L., Bouchaud J. (2004). A non-Gaussian option pricing model with skew. Quant. Financ..

[24] Wang J., Zhang Y. (2018). Geometric Average Asian Option Pricing with Paying Dividend Yield under Non-Extensive Statistical Mechanics for Time-Varying Model. Entropy.

[25] Plastino A.R., Plastino A. (1995). Non-extensive statistical mechanics and generalized Fokker-planck equation. Phys. Stat. Mech. Its Appl..

[26] Tsallis C., Bukman D.J. (1996). Anomalous diffusion in the presence of external forces: Exact time-dependent solutions and their thermostatistical basis. Phys. Rev. Stat. Phys. Plasmas Fluids Relat. Interdiscip. Top..

